# Item response theory analysis of the Utrecht Work Engagement Scale for Students (UWES-S) using a sample of Japanese university and college students majoring medical science, nursing, and natural science

**DOI:** 10.1186/s13104-017-2839-7

**Published:** 2017-10-30

**Authors:** Takashi Tsubakita, Kazuyo Shimazaki, Hiroshi Ito, Nobuo Kawazoe

**Affiliations:** 1 Nagoya University of Commerce and Business, 4-4, Sagamine, Komenoki-cho, Nisshin-shi, Aichi-ken Japan; 2 Department of Nursing College of Life and Health Sciences, Chubu University, 1200 Matsumoto-cho, Kasugai-shi, Aichi-ken Japan

**Keywords:** Academic engagement, The Utrecht Work Engagement Scale for Students, Item response theory, University students

## Abstract

**Objectives:**

The Utrecht Work Engagement Scale for Students has been used internationally to assess students’ academic engagement, but it has not been analyzed via item response theory. The purpose of this study was to conduct an item response theory analysis of the Japanese version of the Utrecht Work Engagement Scale for Students translated by authors. Using a two-parameter model and Samejima’s graded response model, difficulty and discrimination parameters were estimated after confirming the factor structure of the scale.

**Results:**

The 14 items on the scale were analyzed with a sample of 3214 university and college students majoring medical science, nursing, or natural science in Japan. The preliminary parameter estimation was conducted with the two parameter model, and indicated that three items should be removed because there were outlier parameters. Final parameter estimation was conducted using the survived 11 items, and indicated that all difficulty and discrimination parameters were acceptable. The test information curve suggested that the scale better assesses higher engagement than average engagement. The estimated parameters provide a basis for future comparative studies. The results also suggested that a 7-point Likert scale is too broad; thus, the scaling should be modified to fewer graded scaling structure.

**Electronic supplementary material:**

The online version of this article (10.1186/s13104-017-2839-7) contains supplementary material, which is available to authorized users.

## Introduction

Students’ academic engagement has been studied across multiple disciplines [1, 2], including students’ behavioral norms, emotional experiences, and cognitive factors [1–3]. Empirical studies indicate that there is a positive relationships between students’ engagement and their performance [4–6]. These studies have widely used the Utrecht Work Engagement Scale for Students (UWES-S) [7–10]. However, the UWES-S has not been translated into Japanese, nor has it been validated. Moreover, few studies have been conducted on the use of the UWES-S. Therefore, the aim of the current study was to construct the Japanese version of the UWES-S via item response theory.

## Main text

### Participants and procedures

The data for the current study were obtained from previous research on the relationships among students’ engagement, burnout, and other related variables. We used a convenient sampling method. Seven Japanese universities and five colleges were invited to participate. Of the 3401 students contacted, 3280 returned the questionnaires. The participating university students were majoring in medical science, nursing, and natural science. One university and two colleges were national institutions, and six universities and three colleges were private. Students were informed orally and in writing on the front page of the questionnaire that participation was voluntary, and that participation refusal would not negatively impact them. All students were assured that their responses were anonymous. Passive consent was used herein, and participants were informed that by submitting their questionnaire, they were consenting to participate in the study. Before or after class, participants were instructed to complete the questionnaire, which included a cover sheet asking for their age, sex, grade, and other relevant questions. Cases with no answers were excluded. The total number of participants included was 3214 students. This research was reviewed and approved by the ethics committee of Chubu University.

### Measures

The UWES-S [11] was used in the current study (Additional file 1: Appendix). The 14-item version of the UWES-S was selected over the newer 17-item version of the UWES-S [7] owing to how it has been used internationally. Moreover, the preliminary manual of the UWES-S [7] states that the default three factor model of the 17-item version does not fit to the data well (N = 572, Chi square = 59.99, CFI = 0.85, RMSEA = 0.08). It was ultimately converted to an 11-item version (N = 572, Chi square = 92.75, CFI = 0.95, RMSEA = 0.07). The 11-item version is similar to the 14-item version used herein. The original version of the 14-item scale had three factors: vigor (5 items; e.g., “When I’m studying, I feel mentally strong”), dedication (4 items; e.g., “I find my studies to be full of meaning and purpose”), and absorption (5 items; e.g., “Time flies when I’m studying”) [8]. All items are scored on a 7-point Likert scale ranging from 0 = “never” to 6 = “always”.

The Japanese version of the UWES-S used in this study was constructed by using the back-translation technique.

### Analysis

A computer program randomly divided the sample into two groups. Sample 1 included 1607 participants (male = 922, female = 618, others were gender unknown; mean age = 19.85, SD of the age = 2.12); sample 2 included 1607 participants (male = 930; female = 671, others were gender unknown; mean age = 19.79, SD age = 2.11).

First, sample 1 was used for preliminary analysis to examine if item response theory analysis can be applied to the UWES-S. During this stage, the polyserial correlation coefficients were calculated, and an item with a coefficient under 0.20 was removed. Next, confirmatory factor analyses were conducted for the default three-factor structure and for a one-factor structure. The default, three-factor model was the same model that was presented by Schaufeli et al. [8]. The one-factor model was the model where only one latent factor influenced all observed variables. If the one-factor model fit to the data well, item response theory analysis could be conducted without considering the local factors generated by the bi-factor exploratory factor analysis. This process could be followed because this structure is conventionally regarded as one-factor structure. However, in a study by Wefald and Downey, the one-factor model, the two-factor model, and the default three-factor model did not demonstrate satisfactory fit to the data; the authors suggested that the one-factor model was the most parsimonious of the three [12]. Therefore, we implemented a bi-factor exploratory factor analysis as the second best option. In this model, one additional latent factor was installed and the local factors were explored with the data. This additional latent factor is called a general factor. The general factor influences all observed variables, but is not correlated with the local factors that influence the observed variables. If the factor structure with the general factor was confirmed, item response theory analysis could be conducted. Item response theory analysis was conducted using Samejima’s graded response modeling [13] with a two-parameter model. At the end of this stage, items indicating outlier values for each parameter were removed from subsequent analysis.

At the second analytical stage, the resulting items from the first stage were examined using sample 2. The parameters were estimated again and the test information curve represented the amount of test information to consider the characteristics of the scale. It was theoretically described by *I* (*θ*) as follows:

$$I\left( \theta \right) = D^{2} \sum\limits_{j = 1}^{n} {a^{2}_{j} P_{j} \left( \theta \right) \, Q_{j} \left( \theta \right)}$$

where *θ* is the latent trait measured by the scale (i.e., ability parameter), *a* is the discrimination parameter of each item, *P*_*j*_ (*θ*) is item characteristic function, and *Q*_*j*_ (*θ*) is calculated by *1* − *P*_*j*_ (*θ*), and *D* is *1.7*. *P*_*j*_ (*θ*) is calculated as follows:

$$P_{j} \left( \theta \right) \, = \, \left\{ { \, 1 \, + \, exp \, \left[ { - \,1.7a_{j} \left( {\theta - b_{j} } \right) \, } \right] \, } \right\}^{ - 1}$$

where *b* is the difficulty parameter of each item.

All estimation was calculated by the maximum likelihood estimation. To evaluate the fit of each model to the data, we adopted the following indices: (1) the Chi square statistic, (2) the comparative fit index (CFI) [14], (3) the Tucker –Lewis index (TLI) [15], (4) the root mean square error of approximation (RMSEA) [16], and (5) the Akaike information criterion (AIC) [17]. Previous studies indicated that values for CFI and TLI greater than 0.90 indicate acceptable model-data fit [8, 9]. For RMSEA, values less than 0.08 indicate a satisfactory fit, while those greater than 0.10 signify that the model should be rejected [8]. The internal reliability was evaluated by McDonald’s omega coefficient [18].

Items with difficulty parameters more than an absolute value of 6.8 and discrimination parameters out of the range 0.34–3.4 were regarded as outlier values. Analyses were run via the statistical software R, version 3.3.0. The packages used for the analysis are “psych,” “lavaan,” “ltm,” “polycor,” and their related packages. The logistic model in the “ltm” package defines the measurement factor “*D”* as “1.0”.

## Results

The descriptive statistics showed that the item with the lowest mean was item 12 (“When I get up in the morning, I feel like going to class.”) and the item with the highest mean was item 2 (“I find my studies to be full of meaning and purpose.”). The polyserial correlation coefficients ranged from 0.59 to 0.82.

Confirmatory factor analyses showed that the default three-factor model was not satisfactorily supported, and that its fit indices were not necessarily better than those of the one-factor model (one factor model: Chi square = 1721.67, degree of freedom = 77, CFI = 0.86, TLI = 0.84, RMSEA = 0.11, AIC = 70,997.50; default three-factor model: Chi square = 1675.07, degree of freedom = 74, CFI = 0.86, TLI = 0.83, RMSEA = 0.11, AIC = 70,956.90). A bi-factor exploratory factor analysis was conducted, thereby indicating that the model-fit was better than both the one-factor model and the default model (Chi square = 663.99, degree of freedom = 52, RMSEA = 0.086). In this model, three local factors were generated. The model shared some aspects of the default model, but the relations between the factors and items were different. Items 3 and 4 equally loaded on the general factor and the local factor. Other items loaded more on the general factor than the local factors. From the results of the factor analyses, the scale can be analyzed via item response analysis.

The result of the graded response modeling analysis were shown in Table 1; these results indicate that the discrimination parameters of three items were outliers. Therefore, we removed these items in the subsequent analyses.

**Table 1:** Difficulty and discrimination parameters of the UWES-S

Item	Difficulty parameter of category 0 vs 1–6	0–1 vs 2–6	0–2 vs 3–6	0–3 vs 4–6	0–4 vs 5–6	0–5 vs 6	Discrimination parameter
1	− 2.782	− 1.710	− 0.462	1.745	3.491	4.995	0.901
2	− 2.372	− 1.804	− 0.702	0.096	1.454	2.673	1.314
3	− 2.335	− 1.490	− 0.468	0.420	1.591	2.794	1.160
4	− 1.098	− 0.214	0.660	1.411	2.395	3.141	1.582
5	0.015	0.449	0.808	1.467	2.003	2.283	3.622^a^
6	− 0.758	− 0.034	0.589	1.160	1.936	2.922	1.884
7	− 0.155	0.330	0.753	1.356	2.042	2.415	3.539^a^
8	− 1.085	− 0.373	0.315	1.267	2.135	3.012	2.083
9	− 0.427	0.048	0.481	1.043	1.831	2.244	3.452^a^
10	− 0.969	− 0.294	0.210	1.091	1.870	2.564	1.855
11	− 0.941	− 0.305	0.184	0.781	1.487	2.191	1.994
12	0.075	0.575	0.990	1.774	2.253	2.647	2.466
13	− 0.965	− 0.352	0.192	0.997	1.670	2.614	2.314
14	− 0.663	− 0.118	0.342	1.107	1.859	2.515	2.810
Mean	− 1.033	− 0.378	0.278	1.123	2.001	2.786	2.213

^a^Indicates an outlier value

Next, data from sample 2 were used to estimate parameters of the surviving 11 items. The polyserial correlation coefficients ranged from 0.589 to 0.824. The mean difficulty parameters ranged from − 1.542 to 2.857, and the mean of the discrimination parameters was 1.862 (Table 2).

**Table 2:** Difficulty parameters and discrimination parameters of the 11 surviving items

Item	Category 0 vs 1–6	0–1 vs 2–6	0–2 vs 3–6	0–3 vs 4–6	0–4 vs 5–6	0–5 vs 6	Discrimination parameter
1	− 2.659	− 1.714	− 0.650	0.933	2.547	3.713	1.130
2	− 2.763	− 1.880	− 0.898	0.159	1.517	2.754	1.357
3	− 2.452	− 1.665	− 0.661	0.240	1.368	2.597	1.288
4	− 1.465	− 0.493	0.473	1.383	2.381	3.183	1.461
6	− 1.300	− 0.452	0.378	1.184	2.189	3.325	1.420
8	− 1.304	− 0.609	0.082	0.972	1.879	2.689	2.406
10	− 1.243	− 0.553	− 0.005	0.914	1.829	2.493	1.842
11	− 1.334	− 0.656	− 0.127	0.570	1.437	2.133	1.820
12	− 0.324	0.514	1.217	2.409	3.227	3.966	1.410
13	− 1.164	− 0.587	− 0.028	0.720	1.455	2.203	2.990
14	− 0.957	− 0.366	0.156	0.955	1.705	2.368	3.362
Mean	− 1.542	− 0.769	− 0.006	0.949	1.958	2.857	1.862

**Fig. 1:**
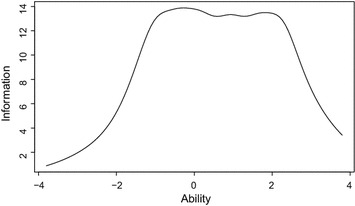
Test information curve for the 11-item version of the Utrecht Work Engagement Scale for Students. “Ability” refers to academic engagement as the latent trait. “Information” refers to the amount of information

The test information curve indicated that these items provide information on the latent trait from − 1.5 to 2.2 (Fig. 1). The omega coefficient of the surviving 11 items was 0.91, suggesting that there was an acceptable internal reliability in this scale.

## Discussion

In the current study, items on the Utrecht Work Engagement Scale for Students were analyzed in the item response theory paradigm. When conducting the item response theory analysis, the one-factor structure required confirmation, and our results suggested that a general factor existed. This was the second best solution to meet the goal of the study: to confirm the one-factor structure of the UWES-S. These results corresponded with Schaufeli et al. where intercorrelations between the default three factors were high, ranging from 0.71 to 0.94 [8].

The parameter analysis indicated that difficulty parameters for each item were generally high, thereby suggesting that participants tended to rate these items from “0” to “2”. The discrimination parameters were also high. Therefore, the items tended to sharply discriminate the degree of engagement. Items with outlier discrimination parameters were items 5, 7, and 9. These items may have confused participants because they ask participants about the perceived strength of one’s positive attitude toward studying; however, the participants were required to select the frequency of these items. The words and phrases of the items—“strong” (item 1), “bursting with energy” (item 7), “strong and vigorous” (item 9), and “enthusiastic” (item 8)—may be difficult to judge in terms of frequency.

Moreover, the difficulty parameters of a rating of 6 versus a rating ranging from 0 to 5 were extremely high. Only a small portion of participants selected a “6”. Thus, it appears that the 7-point structure for this item gave participants too many choices, as suggested by prior research on the UWES-17 [19].

The test information curve suggested that the current 11-item version of the UWES-S provides the most accurate information from *θ* = 0.4 to 2.0. Consequently, this test is suitable for those students who engage more in academic activity than average. This is reflected in the fact that the difficulty parameters of the 11 items were relatively high. Therefore, fewer rating options may be preferable for assessing lower degrees of engagement.

We recommend that the current 11-item version of the UWES-S be used for assessing Japanese students’ academic engagement. However, it is important to note that the Japanese version of the UWES-S should not be shortened to 11 items. Specifically, the items removed herein could be retained by truncating the Likert scale, or refining the translation of the items. Therefore, the 11 items and the removed items require further empirical investigation.

## Limitations

In this study, the 2002 version of the UWES-S was used, not the 2003 version. The difference between the two versions is small; however, additional research is needed to replicate the current findings with the newer 17-item version of the UWES-S. Despite these limitations, the item parameters estimated herein provide useful information for future studies on students majoring in the medical science, nursing, and natural science.

## Additional file

**Additional file 1: Appendix.** Items of the Utrecht Work Engagement Scale for Students.

## Abbreviations

AIC: Akaike’s information criterion
CFI: comparative fit index
RMSEA: root mean square error of approximation
SD: standard deviation
TLI: Tucker–Lewis index
UWES-S: Utrecht Work Engagement Scale for Students
