# Supplementary material for: Item response theory analysis of the Utrecht Work Engagement Scale for Students (UWES-S) using a sample of Japanese university and college students majoring medical science, nursing, and natural science
Source: BMC Res Notes. 2017 Oct 30;10:528. doi: 10.1186/s13104-017-2839-7 (PMC5663090; doi:10.1186/s13104-017-2839-7)
Supplement: Supplementary file 1 — Additional file 1: Appendix. Items of the Utrecht Work Engagement Scale for Students. [file 13104_2017_2839_MOESM1_ESM.docx]

# Appendix: Items of the Utrecht Work Engagement Scale for Students

1. When I'm studying, I feel mentally strong.

2. I find my studies to be full of meaning and purpose.

3. Time flies when I'm studying.

4. I can continue for a very long time when I am studying.

5. My studies inspire me.

6. When I am studying, I forgot everything else around me.

7. When I study, I feel like I am bursting with energy.

8. I am enthusiastic about my studies.

9. When studying, I feel strong and vigorous.

10. I am proud of my studies.

11. I feel happy when I am studying intensively.

12. When I get up in the morning, I feel like going to class.

13. I find my studies challenging.

14. I can get carried away by my studies.
